# Bartter syndrome type III with glomerular dysplasia and chronic kidney disease: A case report

**DOI:** 10.3389/fped.2023.1169486

**Published:** 2023-03-30

**Authors:** Jingqi Liu, Yudi Zhang, Xiaochuan Wu, Yongzhen Li

**Affiliations:** Department of Pediatrics, The Second Xiangya Hospital, Central South University, Changsha, China

**Keywords:** Bartter syndrome, chronic kidney disease, hypokalemia, metabolic alkalosis, glomerular dysplasia

## Abstract

**Background:**

Bartter syndrome (BS) type III is a rare autosomal recessive genetic disease. Its clinical features are polyuria, hypokalemia, hypochloremia, metabolic alkalosis, and hyperreninaemia. A few BS type III can be complicated with chronic kidney disease.

**Case presentation:**

We report a 14-year-old boy with Bartter syndrome caused by a c.1792C > T (*p*.Q598*) mutation in the CLCNKB gene. He was a no deafness and full-term baby, and he had renal dysplasia and chronic kidney disease (CKD). In addition, we summarize all cases of BS type III complicated with CKD.

**Conclusions:**

We report a case of Bartter syndrome complicated by chronic kidney disease caused by a new mutation of CLCNKB. As we all know, BS type IV is usually combined with chronic kidney disease, and BS type III can also integrate with CKD. We don't find BS type III with glomerular dysplasia in the literature. So renal damage in BS type III is not only FSGS; clinicians must also be aware of glomerular dysplasia.

## Background

**Figure 1:**
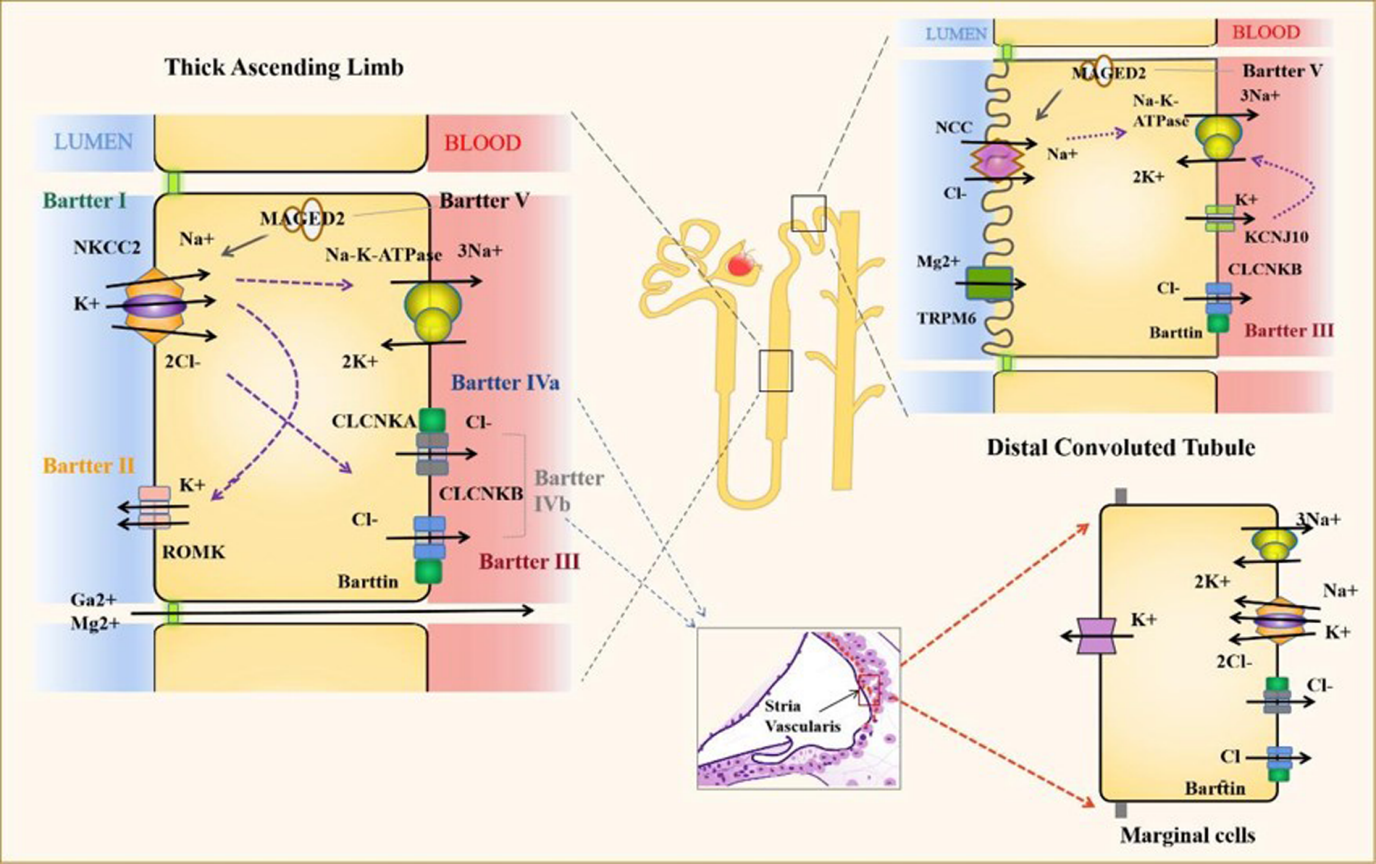
Proteins and channels implicated in the pathogenesis of bartter syndrome. The electrolyte transport of the most important channels for the diseases is represented. Bartter Syndrome IV also shows important ion channels at the stria vascularis. NKCC2, Solute carrier family 12 members 1; ROMK, ATP-sensitive inward rectifier potassium channel 1; CLCNKA, Chloride channel protein ClC-Ka; CLCNKB, Chloride channel protein ClC-Kb; NCC, Solute carrier family 12 members 3; MAGED2, Melanoma-associated antigen D2 and TRPM6,: Transient receptor potential cation channel subfamily M member 6.

Bartter syndrome (BS) is a group of heterogeneous renal tubular diseases with recessive or dominant autosomal inheritance, mainly caused by salt reabsorption disorder. It is primarily characterized by hypokalemia, metabolic alkalosis and secondary aldosteronism and can be combined with hypertension (1, 2). BS is clinically divided into neonatal and classic Bartter syndrome (cBS). The neonatal, also known as the prenatal type, has severe clinical manifestations, such as polyhydramnios, premature delivery, electrolyte, water loss and hypercalciuria (1, 3). Bartter syndrome is divided into 6 types according to genetic classification (Figure 1). BS type I is caused by mutations in the SLC12A1 gene encoding NKCC2, which is responsible for most sodium chloride reabsorption in the apical membrane of epithelial cells (1, 4). BS type II is caused by mutations in the KCNJ1 gene encoding ROMK, which mainly regulates renal K + (4). These two types are usually more severe and can lead to polyhydramnios or premature birth (1). BS type III or cBS is caused by mutations in the Cl voltage-gated channel Kb (CLCNKB) gene, which encodes ClC-Kb and results in loss of its activity, thereby impairing the reabsorption of Cl- by TAL, with less severe clinical manifestations (5).

BS type III is a classic type of Bartter syndrome, and its clinical manifestations are usually not serious. It mainly occurs after infancy, and many forms of CLCNKB gene mutations are associated with it. It is reported in the literature that few patients with BS type III can be complicated with chronic kidney disease (CKD) (5). This article will report a case of BS with glomerular dysplasia and CKD with CLCNKB mutation.

## Case presentation

**Figure 2:**
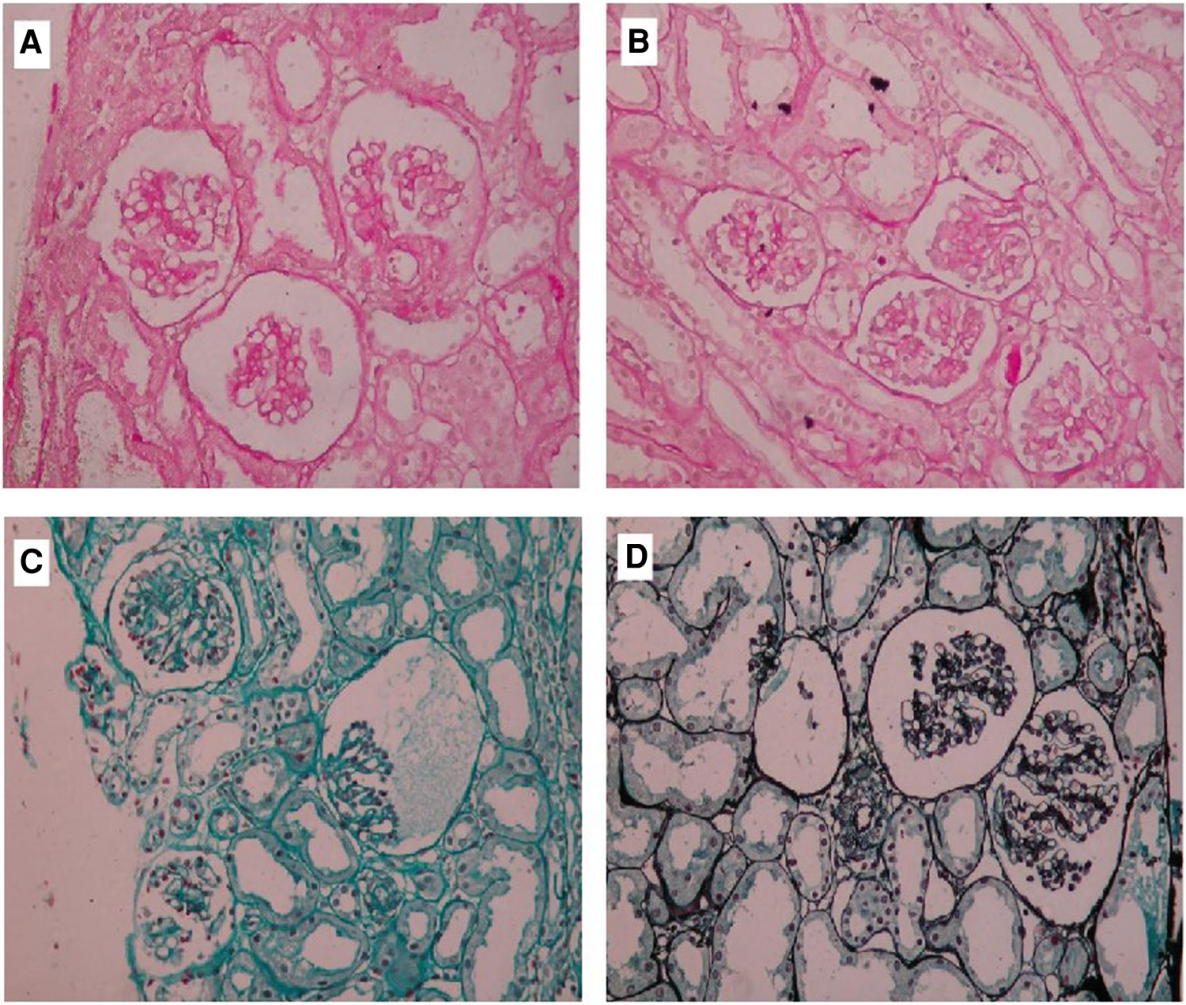
Renal biopsy in a patient with this boy. Photomicrograph of renal biopsy specimen with HE, PAS (**A,B**) stain showed the glomeruli are small and underdeveloped and one juxtaglomerular complex was enlarged and MASSON, PASM (**C,D**) stain showed glomerular basement membrane.

The patient was a 14-year-old Chinese boy. At one age, he was admitted to our hospital for the first time because of polydipsia and polyuria with repeated fever and diarrhoea for 9 months. At that time, his serum potassium was 3.28 mmol/l, pH 7.501, and he had a kidney biopsy (Figure 2). The immunofluorescence examination demonstrated granular staining for Fibrin (++), immunoglobulins (IgA-, IgM-, IgG-) and complements (C3-, C4-).

Unfortunately, his renal function was impaired when the child was 3 years old, and the serum creatinine was 61.0 umol/L. He came to our hospital again because of abnormal renal function for 11 years when he was 14. Physical examination: T was 36.7°C, blood pressure was 100/69 mmHg, HR was 95 bpm, RR was 20 bpm, body height was 160 cm, and body weight was 40 kg. Laboratory examinations are shown in Table 1. Renal colour ultrasonography showed bilateral renal parenchyma enhancement (the left kidney was 79 mm × 33 mm, the right kidney was 79 mm × 31 mm), and thyroid colour ultrasound showed multiple follicular cysts in both lobes of thyroid, diffuse lesions of thyroid parenchyma, slightly larger left parathyroid gland. Chest x-ray and electrocardiogram were normal.

**Table 1:** The laboratory examinations of the proband.

Age (years)	14	15	16	Normal ranges
PH	7.52	-	-	
PO2(mmHg)	188	-	-	
PCO2 (mmHg)	27	-	-	
WBC (10^9^/L)	6.22	4.85	5.50	5.00∼12.00
HGB(g/L)	99	94	111	110∼160
Serum K ^+ ^(mmol/L)	4.10	3.77	2.89	3.50∼5.30
Serum Na ^+ ^(mmol/L)	133.9	137.8	138.7	137.0∼147.0
Serum Cl^−^(mmol/L)	92.0	95.1	91.3	99.0∼110.0
Serum Ca^2 + ^(mmol/L)	2.25	2.43	2.40	2.11∼2.52
Serum P^3 + ^(mmol/L)	1.69	1.49	1.21	0.85∼1.51
Serum Mg^2 + ^(mmol/L)	0.53	0.71	0.69	0.78∼1.27
BUN (mmol/L)	19.93	19.80	18.70	2.90∼7.14
Serum Cr(umol/L)	172.4	228.0	242.0	44.0∼133.0
Blood ATI (ng/L)	28.95	-	-	
Blood ATII (ng/L)	190	-	-	50∼120
ALD (ng/dl)	31.5	-	-	7.0∼30.0
Urine Na (mmol/day)	129.3	110.50	149.40	40.00∼220.00
Urine K (mmol/day)	34.01	31.95	48.34	25.00∼125.00
Urine Cl (mmol/day)	114.00	100.47	132.40	110.00∼250.00
Urine Ca (mmol/day)	2.62	0.11	0.36	2.50∼6.25
Urine Mg (mmol/day)	3.54	2.60	3.58	1.00∼10.50
FT3(pg/ml)	5.07	3.56	-	2.00∼4.40
FT4(pg/ml)	1.44	1.14	-	0.27∼4.20
TSH (uIU/ml)	8.37	8.09	-	0.27∼4.20
PTH (pg/ml)	-	470.20	39.20	18.50∼88.00
Urinary microalbumin(mg/L)	37.72	10	-	0∼30
Urine routine	Normal	Normal	Normal	Normal

After obtaining the consent of both parents, peripheral blood samples of the child and his parents were selected for clinical exon sequencing to analyze the point variation and copy number variations (CNVs). Genomic DNA was interrupted by an ultrasonic crusher, terminal repair, amplification, purification, and other operations to prepare a sequencing library. Specific capture probes (RocheNimbleGen, Madison, WI) were used to hybridize and enrich the DNA sequence of the target region, which included all exon regions, 10 bp intron regions, and known deep intron regions of about 5,000 target genes related to OMIM. Then the second-generation sequencing was carried out on the IlluminaNovaSeq6000 platform. NextGENe software compared the sequencing Reads with the human reference genome (GRCh37/hg19). The high-frequency variation was filtered through the population frequency database of variation (dbSNP, ExAC, gnomAD). The pathogenic mutation sites were evaluated concerning databases such as dbSNP, OMIM, HGMD, ClinVar, and the prediction software such as SIFT, Polyphen2, MutationTaster, and FATHMM were used to predict the conservativeness and pathogenicity of variation.

**Figure 3:**
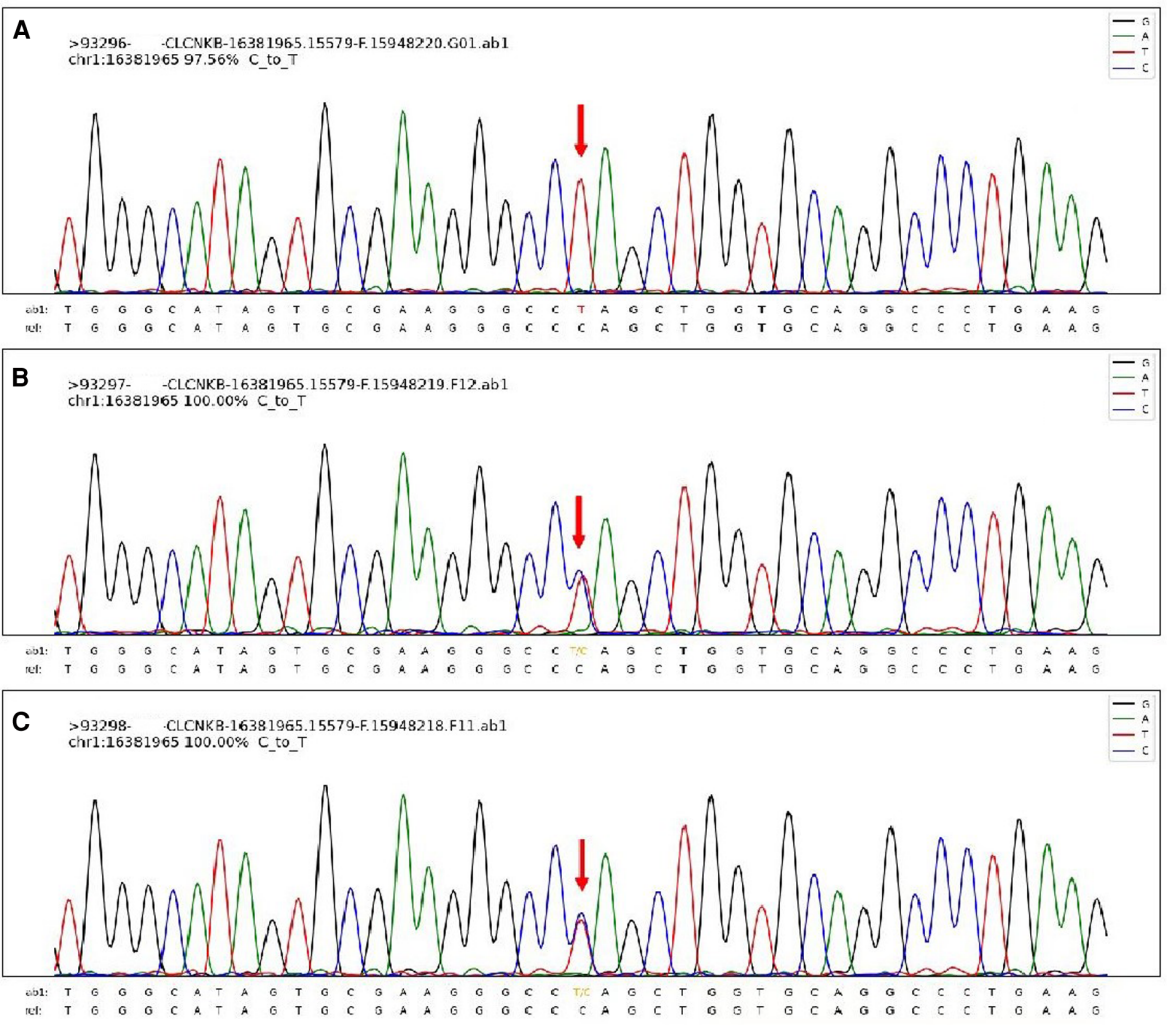
The sanger sequencing map of this patient's family. The patient's homozygous variation CLCNKB gene, which was c.1792C > T (*p*. Q598*) (**A**) and the sanger results of his parents (**B,C**).

Through the above methods, we detected the homozygous variation of the CLCNKB (OMIM 602023 NM_000085) gene, which was c.1792C > T (*p*. Q598*) (Figure 3). This variation is inherited from the unrelated and healthy father and mother, and they are heterozygous. This variation has not been reported in related clinical cases and is a nonsense mutation, which may lead to the termination code of amino acids in protein synthesis in advance. Combined with the clinical manifestations of children, this mutation is pathogenic according to the American College of Medical Genetics and Genomics (ACMG) mutation classification guidelines. In addition, the thyroid function of this child is abnormal, which may be related to the variation of TG gene c.4082G > A (*p*.R1361H) and c.7753C > T (*p*.R2585W) in this child, which is from the father and mother respectively.

The child has been taking spironolactone and potassium chloride to supplement potassium throughout the disease to maintain serum potassium. However, the child's renal function was still damaged. At discharge, his serum potassium was 2.89 mmol/L, the creatinine was 242.0 mmol/L, and the electrolyte indicated hypochloremia and hypomagnesemia.

## Discussion

More than one million Bartter syndrome (BS) cases have been reported. BS is an autosomal recessive renal tubular disease caused by mutations in genes encoding ion channels or transporters. According to molecular genetics, it can be divided into Bartter syndrome type I-V (4). The clinical manifestations of Bartter syndrome are complex and diverse, among which hypokalemia is the main. BS type I and type II are usually severe, which can lead to polyhydramnios during pregnancy and premature delivery (6). BS type III, also known as classic Bartter syndrome, usually has no serious clinical manifestations and often occurs in infancy (5). BS type IV is traditionally associated with hearing loss, mainly with renal impairment (7).

BS type III or cBS is caused by a mutation of the CLCNKB gene, which encodes ClC-Kb and causes its activity loss. ClC-Kb is a member of the ClC chloride channel family, is expressed in the coarse ascending branch, distal convoluted tubule and cortical collecting tubule of the Henle loop, and regulates the reabsorption of Cl in renal tubules (5). Inactivated ClC will reduce the reabsorption of chloride and sodium in renal tubules, and the loss of NaCl and water will activate the renin-angiotensin-aldosterone system (RAAS), which will lead to the loss of potassium and renal fibrosis (8).

In our case, according to the clinical manifestations and gene detection results, the diagnosis was consistent with Bartter syndrome type III. BS type III has mild clinical symptoms and less chance of involving renal function. As we know, the renal pathology of BS can be membranous proliferative glomerulonephritis, interstitial glomerulonephritis, renal calcification, and other pathological changes, showing little or no abnormality in glomeruli or renal tubules, hyperplasia and hypertrophy of juxtaglomerular complex are the main pathological changes of this disease (9, 10). However, this child has renal dysfunction. According to renal biopsy results, the child has juxtaglomerular complex hypertrophy, which is in line with BS renal dysfunction. The child still has glomerular dysplasia and has not used nonsteroidal anti-inflammatory drugs (NSAIDs) during the treatment. We don't find BS type III with glomerular dysplasia in the literature. Renal dysplasia may lead to CKD and, with time, progress to end-stage renal disease (ESRD) (11). Therefore, we speculate that the boy's renal dysfunction may be related to his glomerular dysplasia.

A few literatures reports on cases of BS type III complicated with chronic kidney disease (Table 2).

**Table 2:** Genotype and clinical features of each cBS and CKD.

Author	Year	Age at Diagnosis	Sex	Nucleotide (cDNA)	Protein	Type of mutation	Serum creatinine (umol/l)	GFR^1^ (ml/min per 1.73 m^2^)	Reference
Schachter et al.	1998	1y	M	-	-	-	107 (16y)	94.8(proteinuria 1.2∼3.4 g/d)	(12)
								39	
		3.5y	F	-	-	-	141 (8y)		
Schurman et al.	2000	2m	-	-	-	-	55.0 (2m)	88	(13)
Lin et al.	2009	1y	M	c.1520G > A	G470E	Missense	176.8 (12y)	-	(8)
Seys et al.	2017	7y	F	-	-	Large deletions	-	80	(5)
		2m	M	-	-	Missense	-	81	
		3m	M	-	-	Large deletions	-	82	
		0.16y	M	-	-	Large deletions	-	27	
		0.5y	F	-	-	Missense	-	CKD5	
		8y	M	-	-	Missense	-	13	
		0.16y	F	-	-	Large deletions	-	CKD5	
Zhang et al.	2018	1y	F	del Exon2-3	-	-	175.0 (15y)	35	(9)
Zhu B et al.	2019	4m	F	c.1969 del G	G566fs	Frameshift	175.0 (14y)	43	(14)
				del Exon2∼3	-	-			
Le et al.	2020	13y	M	-	S12A	Missense	124.0 (13y)	61	(10)
					G192T	Nonsense			

GFR^1^, glomerular filtration rate.

In 1998, *Schachter* et al. said that two children with cBS had renal impairment, but their renal impairment occurred after using NSAIDs, and their renal function was restored after stopping using NSAIDs (12). Therefore, scholars think that it may be due to the direct cytotoxic effect of NSAIDs and the renal ischemia caused by inhibiting vasodilation and automatically regulating prostaglandins, which leads to functional impairment (12). *Lin* et al*.* has the same view. He reported a 17-year-old patient diagnosed with classic BS complicated with renal impairment in 2009. The boy started to get sick at the age of 9 months, and his renal function decreased progressively. At the age of 12, the creatinine was 2.0 mg/dl, and at the age of 17, the creatinine reached 6.3 mg/dl. After stopping indomethacin, his serum creatinine was decreased (8). Therefore, the author speculated that the long-term use of NSAIDs caused renal damage. Two scholars have concluded that some studies have shown that the secretion of urinary prostaglandin E2 (PGE2) in BS patients caused by chloride channel defects is reduced, which will not benefit from the long-term use of NSAIDs (8, 15).

Of course, not all renal function damage is caused by NSAIDs. In 2001, *Schumann* et al*.* reported five African American children with BS, one of whom was diagnosed with cBS at 2 months old. His glomerular filtration rate (GFR) reached 88 ml/min/1.73 m^2^, but the author did not analyze the causes of renal dysfunction (13). In the article of *Seys*, 115 patients with CLCNKB mutation were analyzed, and 51 children were diagnosed with cBS, of which seven had different degrees of renal impairment (5). Considering the birth weight, diagnosis age, long-term NSAIDs treatment, persistent hypokalemia and other renal abnormalities, the author did not point out the factors of renal dysfunction in these children (5).

In addition, the changes in children's kidney diseases will also affect their kidney function. In 2018, *Zhang* et al*.* reported that a child with cBS was admitted to the hospital because of general weakness, polydipsia, polyuria, and repeated vomiting for more than 10 years, accompanied by irritability, hyperhidrosis, loss of appetite, and weight loss (9). At admission, her serum creatinine was 175 umol/L, and the GFR was 35 ml/min/1.73 m^2^. Unlike the previous BS children, her kidney biopsy was consistent with the kidney injury of Bartter syndrome. Pathology showed that focal segmental glomerulosclerosis (FSGS) and peribulbar complexes proliferate around some segmental sclerosis areas. Her gene result was heterozygosity loss of exons 2 and 3 of the CLCNKB gene (9). In 2019, *Zhu* et al*.* reported a 15-year-old child with cBS whose creatinine clearance rate was 43 ml/min/1.73 m^2^. His renal puncture suggested focal segmental glomerulosclerosis (14). Under an electron microscope, focal segmental glomerulosclerosis, glomerular basement membrane thickening, immune complex deposition in the mesangial matrix, vacuolar degeneration of renal tubular epithelial cells, renal interstitial fibrosis and inflammatory cell infiltration were observed (14). The author puts forward two hypotheses about the changes in renal function in this child: one possibility is that chronic stimulation of RAAS increases the response of ATII to chronic renal dysfunction caused by salt loss nephropathy; the other possibility is that long-term hypokalemia may lead to hypertrophy and renal fibrosis by activating transforming growth factor *β* (TGF-*β*), so the scholar thinks that the renal changes in this child are caused by BS (14).

*Le* et al*.* reported a 13-year-old Vietnamese boy diagnosed with cBS. His creatinine was 124umol/l, and his GFR was 61 ml/min/1.73 m^2^ (10). Because there are few cases of cBS complicated with renal insufficiency, his genetic detection was improved. Two mutations were found in CLCNKB, including a replacement of serine at position 12 (Ser-12) of exon 2 by alanine (Ser12 Ala) and a replacement of glutamine at position 192 of exon 6 by a stop codon (Glu 192Ter- nonsense mutation) (10). His renal pathology suggests that FSGS and the author think it is secondary to BS. After 6 months of treatment with enalapril (0.17 mg/kg/ day), the normal plasma sodium and potassium concentrations are maintained, and the renal function has not deteriorated further (10). He also said long-term enalapril treatment might be a good choice for patients with classic Bartter syndrome with CKD (10).

*Tamagawa* et al. think chronic renal failure can increase serum potassium levels, decrease urinary calcium reabsorption, and induce metabolic acidosis (16). Likewise, long-term hypokalemia can induce vacuolation of proximal and distal renal tubules and stimulate ammonia production. A high concentration of NH4+/NH3 can cause complement activation, which can start immune cells to enter the stroma (8). The low volume, high renin and high aldosterone in BS may be the related factors of renal damage. As we all know, aldosterone can promote myocardial fibrosis, and some studies have shown that aldosterone can also cause renal fibrosis in hypertensive rats (17). ATII can also promote renal fibrosis in rats and induce the expression of TGF-*β* (18).

In addition, our child has other symptoms besides renal function damage, such as thyroid dysfunction and hypomagnesemia. However, no literature has been found to report the relationship between thyroid function and BS, and we think that the abnormal TF of this child may be caused by its genes instead of BS. Hypomagnesemia mainly occurs in GS, but this abnormality can't completely distinguish GS from BS (1).

Currently, BS treatment focuses on maintaining normal serum electrolytes and controlling the increase of prostaglandin E2 (19). Commonly used drugs are NSAIDs, potassium-preserving diuretics, RAAS inhibitors and growth hormone (GH) (1). NSAIDs are currently the first-line drugs to treat and control the increase of PGE2, even though long-term use may lead to kidney and gastrointestinal damage. NSAIDs should be avoided as much as possible for children with BS and renal injury. Recent studies have shown that acetazolamide and progesterone have specific curative effects in relieving metabolic alkalosis and hypokalemia. Still, the popularization of these two drugs needs further clarification through experiments (20, 21).

In conclusion, this case reports a new gene mutation about BS, which increases the understanding of the molecular mechanism of this disease. At the same time, the boy has glomerular dysplasia. We need to pay attention to children with BS type III with renal damage and not only FSGS but also sometimes glomerular dysplasia. We also summarized renal insufficiency reported in the literature. These can let clinicians know more about the kidney function damage caused by BS type III, and if necessary, improve renal puncture and genetic testing to determine the cause. We should also pay attention to avoid using NSAIDs for BS with kidney damage.

## Ethics statement

Written informed consent was obtained from the participant/patient(s) for the publication of this case report.

## Author contributions

JL was a first-year resident and wrote the manuscript. YZ collected the patient data. XW and YL analyzed and interpreted the data. All authors contributed to the article and approved the submitted version.

## Conflict of interest

The authors declare that the research was conducted in the absence of any commercial or financial relationships that could be construed as a potential conflict of interest.
